# Inhibition of the glutaredoxin and thioredoxin systems and ribonucleotide reductase by mutant p53-targeting compound APR-246

**DOI:** 10.1038/s41598-018-31048-7

**Published:** 2018-08-23

**Authors:** Lena Haffo, Jun Lu, Vladimir J. N. Bykov, Sebastin S. Martin, Xiaoyuan Ren, Lucia Coppo, Klas G. Wiman, Arne Holmgren

**Affiliations:** 1 Division of Biochemistry, Department of Medical Biochemistry and Biophysics, Karolinska Institutet, SE-171 77 Stockholm, Sweden; 2 Department of Oncology-Pathology, Cancer Center Karolinska (CCK), Karolinska Institutet, SE-171 76 Stockholm, Sweden; 3 School of Pharmaceutical Sciences, Southwest University, 400715 Chongqing, China

## Abstract

The tumor suppressor p53 is commonly inactivated in human tumors, allowing evasion of p53-dependent apoptosis and tumor progression. The small molecule APR-246 (PRIMA-1^Met^) can reactive mutant p53 in tumor cells and trigger cell death by apoptosis. The thioredoxin (Trx) and glutaredoxin (Grx) systems are important as antioxidants for maintaining cellular redox balance and providing electrons for thiol-dependent reactions like those catalyzed by ribonucleotide reductase and peroxiredoxins (Prxs). We show here that the Michael acceptor methylene quinuclidinone (MQ), the active form of APR-246, is a potent direct inhibitor of Trx1 and Grx1 by reacting with sulfhydryl groups in the enzymes. The inhibition of Trx1 and Grx1 by APR-246/MQ is reversible and the inhibitory efficiency is dependent on the presence of glutathione. APR-246/MQ also inhibits Trxs in mutant p53-expressing Saos-2 His-273 cells, showing modification of Trx1 and mitochondrial Trx2. Inhibition of the Trx and Grx systems leads to insufficient reducing power to deoxyribonucleotide production for DNA replication and repair and peroxiredoxin for removal of ROS. We also demonstrate that APR-246 and MQ inhibit ribonucleotide reductase (RNR) *in vitro* and in living cells. Our results suggest that APR-246 induces tumor cell death through both reactivations of mutant p53 and inhibition of cellular thiol-dependent redox systems, providing a novel strategy for cancer therapy.

## Introduction

The p53 tumor suppressor plays a key role in the protection against tumor growth^1,2^. However, TP53 is inactivated in a major fraction of human tumors, mainly by missense mutations in the DNA-binding core domain of p53^3,4^, leading to loss of DNA binding and p53-dependent transactivation of target genes such as p21, Bax and Puma. The central role of p53 in stress-induced apoptosis, the frequent TP53 mutations in tumors, and the high levels of mutant p53 in tumor cells have stimulated efforts to develop novel cancer therapy by reactivating mutant p53^5^. Several small molecules that target mutant p53 have been identified through various strategies, including PRIMA-1 and APR-246 (PRIMA-1^Met^)^6,7^, 3-Benzoylacrylic acid^8^, PK7088^9^, NSC319726 (ZMC1)^10^, stictic acid^11^ and 2-sulfonylpyrimidines such as PK-11007^12^. APR-246 has been tested in a first-in-man phase I/II clinical trial in patients with hematological malignancies or prostate cancer and shown promising results^13^, and is currently tested in a phase I/II clinical trial in patients with high-grade serous ovarian cancer (www.clinicaltrials.gov).

PRIMA-1 and APR-246 are both converted to methylene quinuclidinone (MQ), a Michael acceptor that binds covalently to cysteine (Cys) residues in p53, and this binding is sufficient for p53 reactivation^14^. APR-246/MQ has also been shown to inhibit thioredoxin reductase (TrxR) *in vitro* and in living cells^15^. In addition, APR-246/MQ binds to and depletes cellular glutathione^16,17^.

The ability of MQ to bind to cysteines raised the question as to whether it could bind to thioredoxin 1 (Trx1) and/or glutaredoxin 1 (Grx1) and inhibit these proteins. Trx1 belongs to the Trx1 system, which includes Trx1, TrxR1 and nicotinamide adenine dinucleotide phosphate (NADPH)^18,19^. Grx1 is part of the Grx1 system, consisting of Grx1, glutathione (GSH), glutathione reductase (GR) and NADPH^20^. These systems are present in all mammalian cells^19,20^, and are required for the activity of ribonucleotide reductase (RNR) which provides deoxyribonucleotides for DNA replication and repair. Each turnover of the enzyme results in a disulfide in the buried active site which has to be reduced^21^. Trx and Grx systems have been recognized as the primary electron donors to reduce RNR^22^ which is often induced in tumor cells^23^. The Trx system also reduces peroxiredoxins which control reactive oxygen species (ROS) and hydrogen peroxide levels^19^. Trx1 and Grx1 regulate apoptosis signal-regulating kinase1 (ASK1) by binding and inactivating the enzyme only in their reduced form^24^. Many transcription factors are controlled by reduced Trx1 and Grx1, including p53, nuclear factor-κB (NF-κB), and Activator protein 1 (AP-1)^19,25–27^.

The pro-inflammatory and anti-apoptotic properties of the Trx1 and Grx1 systems, as well as their redox capabilities, make them important for tumor cell survival. Tumor cells, in general, have a highly active glucose metabolism due to rapid proliferation, resulting in increased production of ROS^23^. The Trx1 and Grx1 systems are thus commonly upregulated in cancer cells to counteract the oxidative stress as a result of increased ROS production, which otherwise can lead to apoptosis or necrosis^23^. Trx1 expression was inversely correlated to the p53-dependent regulation of tumor growth in breast cancer^28^. Inhibition of Trx1 and Grx1 could, therefore, have pro-apoptotic effects in cancer cells^23,29^.

Here we investigated the potential interactions between APR-246/MQ and Trx1 and Grx1, and found that reaction of MQ with both Trx1 and Grx1 was associated with loss of free thiols in both proteins. Mass spectrometry confirmed that the inhibitory effect of MQ was due to its binding to thiols. The effect on Trxs was also evident in tumor cells where both Trx1 and Trx2 were modified. Moreover, we found that APR-246/MQ is a potent inhibitor of RNR, both *in vitro* and in living cells. These results provide further evidence for a potent effect of APR-246 on cellular redox regulation.

## Materials and Methods

### Proteins and reagents

Recombinant human Trx1 and Grx1, rat recombinant TrxR and polyclonal goat anti-Trx1 antibody were obtained from IMCO Corporation, Sweden (www.imcocorp.se). Anti-Trx2 was from Santa Cruz Biotechnology (Santa Cruz, CA, USA). Recombinant yeast GR and all other chemicals and reagents were of analytical grade and obtained from Sigma-Aldrich. APR-246 and MQ were provided by Aprea Therapeutics AB (Stockholm, Sweden). Before using, APR-246 was freshly dissolved in DMSO and preheated at 90 °C for 10 min, and then diluted into the desired concentration.

### Cells and Cell culture

Human Saos-2 osteosarcoma cells are p53 null. The Saos-2-His-273 subline carries exogenous His-273 mutant p53 which is amenable to reactivation by APR-246^6^. Cells were cultured in Iscove’s modified Dulbecco’s medium (IMDM) with 4 mM L-glutamine and 25 mM HEPES, and supplemented with 10% fetal bovine serum.

### Assessment of Grx1 system activity *in vitro*

Grx activity assessment is based on the method of Coppo *et al*.^30^. Briefly, human recombinant Grx1 (0.1 µM) was pre-reduced in a 96-well microtiter plate for 30 min at 37 °C in the presence of 0.25 mM NADPH, 50 nM yGR, 50 µM GSH and 5 µg/mL BSA in KE buffer (0.1 M Potassium phosphate, pH 7.5 and 1 mM EDTA) in final concentrations. Indicated concentrations of APR-246 were added and after 60 min incubation the reaction was started by adding 20 µM Eosin-GSH labeled BSA (E-GS-BSA). Fluorescence was recorded at 545 nm emission after 520 nm excitation for 45 minutes using a Victor3 fluorescence plate reader (PerkinElmer, USA). The activity of Grx1 was obtained through calculation of the increasing fluorescence intensity over time due to the release of eosin-GSH.

### Assessment of Trx1 system activity *in vitro*

The method was based on a fluorescent microtiter method^31^. Human recombinant Trx1 (0.1 µM) was prereduced in a 96-well microtiter plate for 30 min at 37 °C in the presence of 50 nM TrxR and 0.25 mM NADPH in TE buffer (100 mM Tris, pH 7.5 and 10 mM EDTA) in final concentrations. Indicated concentrations of APR-246 were added and after 60 min incubation, 6 µM fluorescein isothiocyanate coupled to insulin (FITC-insulin) was added to start the reaction^31^. Fluorescence was recorded at 520 nm emission after 485 nm excitation for 30–45 minutes using a Victor3 fluorescence plate reader (PerkinElmer, USA). The activity of Trx1 was obtained by calculating the increasing fluorescence intensity over time within the linear area of the curve.

### Assessment of reduced Trx1 or Grx1 *in vitro*

Trx1 and Grx1 were reduced with 10 mM dithiothreitol (DTT) for 1 hour at 37 °C, and desalted using a NAP-5 column (GE Healthcare, Uppsala, Sweden) and eluted with TE buffer for Trx1 or KE buffer for Grx1. The absorbance of each fraction was measured with a spectrophotometer and the concentration was calculated for A_280_ with a molar extinction coefficient of 8000 M^−1^ cm^−1^ for hTrx1 and 3230 M^−1^ cm^−1^ for hGrx1. A full reduction of Trx1 and Grx1 was determined with 6 M guanidine hydrochloride in 0.1 M Tris-Cl, pH 7.5 and 1 mM 5,5′-dithiobis(2-nitrobenzoic acid) (DTNB) as described^32^. Absorbance was measured at 412 nm and the amount of free thiols was calculated with the molar extinction coefficient for DTNB (13600 M^−1^ cm^−1^). Fully reduced protein was incubated with indicated concentrations of APR-246 for one hour at 37 °C and desalted with Amicon Ultra Centrifugal Spinners with 3000 cutoff for 30 min at 14000 g to remove excess compound. Finally, the activities of Trx1 and Grx1 was determined with the fluorometric methods as described above.

### Reversibility and thiol dependence of APR-246 binding to Trx1 and Grx1

Reduced Trx1 and Grx1 were incubated with 240 and 1000 µM APR-246, respectively for one hour at 37 °C prior to assessment of protein thiol content with DTNB as described above. MQ-modified Trx1 was then incubated for one hour at 37 °C with either 10 mM DTT or 50 nM TrxR plus NADPH. Grx1 treated with preheated APR-246 was incubated with 10 mM DTT for one hour at 37 °C. After incubation, free DTT and NADPH were removed by desalting and protein thiol content was assessed once again to identify restoration of thiols in Trx1 and Grx1. Untreated Trx1 and Grx1 were used as controls.

### Mass spectrometry analysis

Pre-reduced recombinant hTrx1 and hGrx1 (50 µM) were incubated with 0.1 and 0.5 mM MQ, respectively, for one hour at 37 °C. Modified proteins were desalted with C4 ZipTip and then crystallized on a matrix-assisted laser desorption/ionization (MALDI) target plate with MALDI matrix prepared through dissolving 10 mg Sinapinic acid in 75% acetonitrile and 0.1% trifluoroacetic acid (TFA). MQ-modified Trx1 and Grx1 were identified using MALDI-TOF mass spectrometry (Voyager DE-Pro, AB SCIEX). Each spectrum was the result of 50 laser shots and myoglobin was used for external calibration.

### Redox Western blot analysis of cellular Trx1 and Trx2

Detection of Trx1 and Trx2 redox states was carried out as described^33^, using p53 null Saos-2 and mutant p53-expressing Saos-2-His-273 cells. 19800 cells/cm^2^ were plated on 15 cm dishes for 18 hours prior to 24 hours of treatment with 65 µM APR-246. The cells were then harvested for Trx redox state detection.

### Total cellular glutathionylation detection

Cells were seeded into 6-well plates at 3 × 10^5^ cells per well, and treated with different concentrations of APR-246 the following day. After 24 hours treatment, cells were washed and harvested in cold PBS and lysed in lysis buffer (25 mM Tris·HCl, pH 7.5, 100 mM NaCl, 2.5 mM EDTA, 2.5 mM EGTA, 20 mM NaF, 1 mM Na3VO4, 20 mM sodium ß-glycerophosphate, 10 mM sodium pyrophosphate, 0.5% Triton X-100) containing protease inhibitor cocktail (Roche) and 50 mM iodoacetamide (IAM, Sigma-Aldrich). After centrifugation, 25 µg of total proteins from the supernatant were separated by SDS-PAGE under the non-reducing condition and probed with an anti-glutathione antibody (VIROGEN Corporation). Anti-GAPDH (Santa Cruz) antibody was used as loading control.

### Assay for ribonucleotide reductase activity *in vitro*

RNR was reconstituted by mixing recombinant R1 and R2 proteins. Activity was assayed following the conversion of [^3^H]CDP into [^3^H]dCDP. The reaction was initiated by adding reaction mixture containing 40 mM HEPES buffer, pH 7.6, 2 mM ATP, 10.6 mM MgCl_2_, 200 mM KCl, 20 µM FeCl_3_, 5 mM DTT and 0.3 mM [^3^H]CDP (5548 cpm/nmol) in a final volume of 50 µl. Incubation was carried out at 37 °C for 30 min. The reaction was terminated by the addition of 0.5 ml of 1 M HClO_4_ and 50 µl of 20 mM dCMP was added as a carrier and placed in boiling water for 10 min to hydrolyze CDP and dCDP into CMP and dCMP. The protein precipitate was pelleted by centrifugation, the dCMP/[^3^H]dCMP formed was isolated by ion exchange chromatography on Dowex-50 W columns, and the amount of radioactivity was quantified by liquid scintillation counter^33^. RNR was incubated with different concentrations of APR-246 or MQ for 30 min at 37 °C followed by the addition of the reaction mixture.

### Assay for ribonucleotide reductase activity in cultured cells

Saos-2 and Saos-2-His-273 cells were treated with 12.5, 25, and 50 µM of APR-246 for 24 hours. The cells were scraped, spin down, and resuspended in HEPES buffer (100 mM HEPES, pH 7.6, 15 mM magnesium acetate and 10 mM DTT) with freshly added protease inhibitor (Roche Applied Science). The lysate was passed through five cycles of freeze-thaw and sonicated; debris was removed by centrifugation (13,000 rpm, 20 min, 4 °C) and the supernatant was assayed. Protein concentration was determined by the Bio-Rad protein assay. Assays were performed according to the method described by Lepoiver *et al*. [47] with some modifications using [^3^H]CDP as a substrate, ATP as effector and DTT as a reductant. The cell extracts were added to a series of tubes with/without a constant amount of recombinant R1 and R2 and incubated with the [^3^H]CDP buffer (100 mM HEPES, pH 7.6, 15 mM magnesium acetate, 10 mM ATP, 200 µM CDP, 5 mM DTT, 0.5 mM [^3^H]CDP (Vitrax, Placentia CA USA, 5548 cpm/nmol) for 30 min at 37 °C in a final volume of 90 µl. The reaction was terminated by the addition of 1 M HClO_4_, and 50 µl of dCMP was added as a carrier. The samples were heated to 90 °C for 10 min in boiling water and the protein precipitate formed were pelleted by centrifugation. The dCMP/[^3^H]dCMP formed was isolated by ion exchange chromatography on Dowex-50 W columns, and the amount of radioactivity was quantified by liquid scintillation counter^33^.

### Data analysis

Data were analyzed with GraphPad Prism 5 software (GraphPad Software, San Diego, CA, USA) and Quantity One 1-D analysis software (Bio-Rad, CA, USA).

## Results

### Effects of GSH on the inhibition of glutaredoxin and thioredoxin systems by APR-246

**Figure 1:**
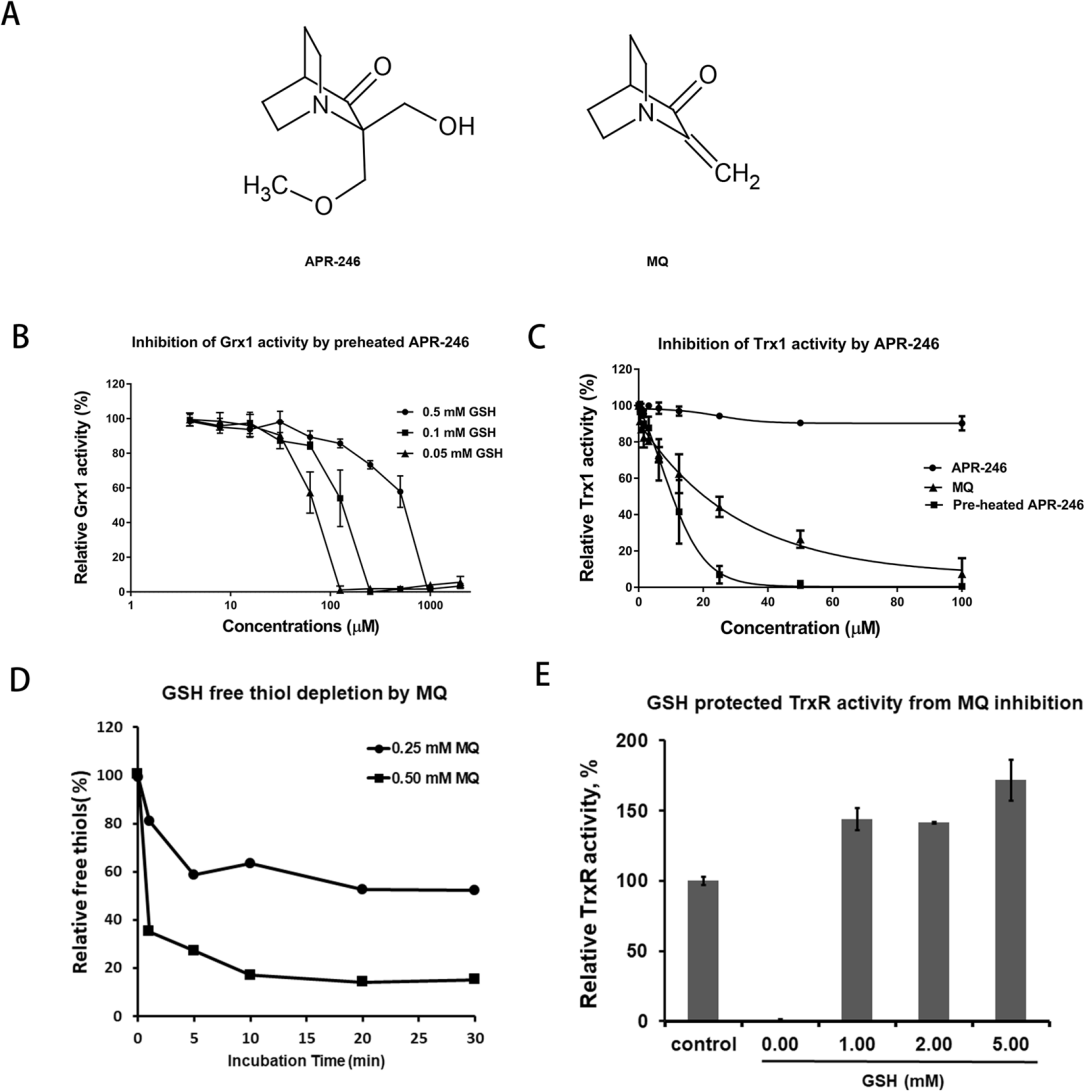
Inhibition of the GSH-Grx system and Trx system by APR-246. (**A**) The chemical structures of APR-246 and MQ. (**B**) The GSH-Grx system (0.25 mM NADPH, 50 nM yeast GR, 50–500 µM GSH, 0.1 µM Grx1) was incubated with pre-heated APR-246 in a 96-well microtiter plate for 60 min. Grx1 activity was assayed by addition of 20 µM E-GS-BSA. Data are shown as mean ± SD (n = 3). (**C**) Inhibition of Trx system by APR-246, preheated APR-246 and MQ. After incubation of the Trx system (0.1 µM Trx1, 50 nM TrxR, and 0.25 mM NADPH) with APR246, preheated APR-246, and MQ for 60 min, 6 µM FITC-insulin was added to measure Trx activity. Results are shown as the means ± SEM of three experiments. (**D**) Time course of interaction of GSH with MQ. GSH (0.5 mM) was incubated with 0.25 and 0.5 mM MQ with various time points and unreacted free thiols in GSH were determined by titration with 2 mM DTNB. The reaction was shown to be a second order with a rate constant (k_2_) of 21.6 M^−1^ s^−1^. (**E**) Recombinant rat TrxR (0.5 µM) was reduced by 200 µM NADPH for 5 min and then incubated with 200 µM MQ in the absence or presence of GSH at room temperature overnight. The GSH was then removed by Microspin^TM^ G-25 columns and TrxR activity was measured by a DTNB reduction assay. TrxR without the treatment with MQ was used as control. Data are shown as mean ± SD (n = 2).

In order to stimulate conversion of APR-246 to MQ, we preheated APR-246 at 90 °C for 10 min. The effect of preheated APR-246 on the reducing activity of the Grx system was investigated using a novel fluorescence-based activity assay with Eosin-GSH labeled BSA (E-GS-BSA) as a substrate. Increasing inhibition of Grx1 was observed with decreasing concentrations of GSH (Fig. 1B). APR-246 had no significant inhibitory effect on the Trx system, while preheated APR-246 and MQ displayed an effective inhibition (Fig. 1C).

The results from the Grx activity assays prompted further examination of the reactivity of MQ with GSH. When 0.5 mM GSH was incubated with MQ and the thiol content assessed with DTNB, a reaction with a rate constant of 21.6 M^−1^ s^−1^ was observed (Fig. 1D). In a second experiment, the reactivity of NADPH-reduced TrxR, known to be inactivated by MQ (15), was tested with and without GSH. A complete inactivation of TrxR was obtained in the absence of GSH, whereas 1 mM GSH fully protected the enzyme (Fig. 1E). Thus, MQ reacts with GSH, in agreement with recent studies^16^.

### Direct reaction of preheated APR-246 with Trx1 and Grx1

**Figure 2:**
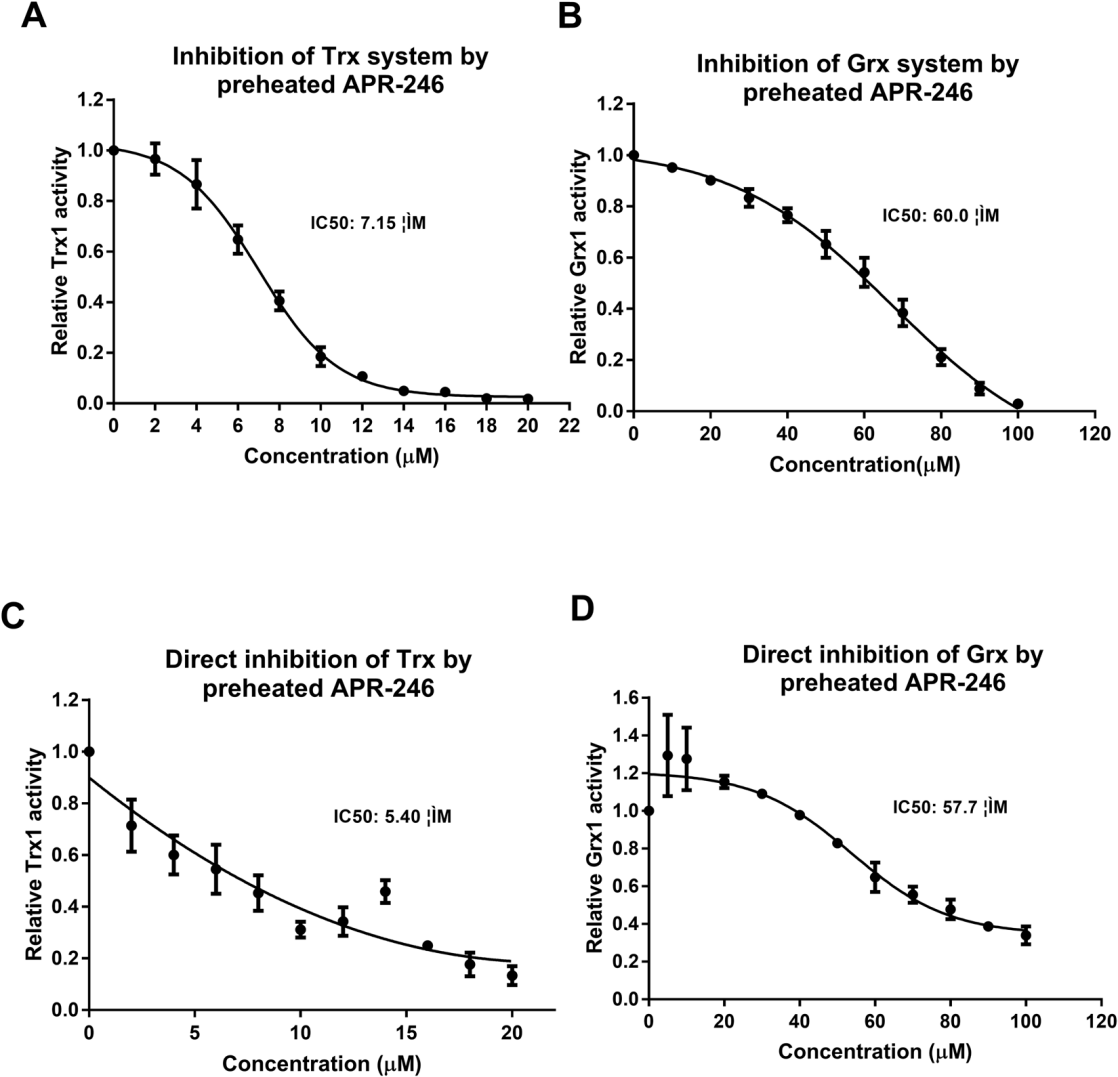
Comparison of the inhibition of the hTrx1 and hGrx1 systems with the inhibition of individual Trx1 and Grx1 by APR-246. (**A**) Inhibition of the Trx system by preheated APR-246. The thioredoxin system (50 nM TrxR1, 0.1 µM Trx, and 0.25 mM NADPH) was incubated with preheated APR-246 (2–20 µM) for 60 min. Trx activity was then assessed by the FITC-insulin coupled method to measure IC_50_ value. Results are shown as means ± SD of three experiments. (**B**) Inhibition of the Grx system by preheated APR-246. The GSH-Grx system (0.25 mM NADPH, 50 nM yeast GR, 50 µM GSH 0.1 µM Grx1) was incubated with pre-heated APR-246 (10–100 µM) for 60 min. Grx1 activity was then assessed by the E-GS-BSA coupled method. Results are shown as means ± SEM of three experiments. Inhibition of Trx1 (**C**) and Grx1 (**D**) by preheated APR-246. Trx1 and Grx1 were prereduced with 10 mM DTT and desalted prior to 60 min incubation. The mixtures were then added onto 96-well plates along with the remaining components of the respective systems to measure the activity as described above. Results are shown as means ± SEM of three experiments.

We then investigated if preheated APR-246 acted as an inhibitor of Trx1 and Grx1 specifically or through interaction with other components of the two systems, which was recently shown for TrxR1^15^. The IC_50_ of the inhibition of the Trx system (Fig. 2A) and the Grx system (Fig. 2B) by preheated APR-246 was 7.15 and 60.0 µM, respectively. To detect the direct reaction of Trx1 and Grx1 with preheated APR-246, DTT-prereduced Trx1 and Grx1 were incubated with preheated APR-246. The remaining components of the respective systems were then added to the mixture to measure the activity. Preheated APR-246 showed stronger inhibition of Trx1 (Fig. 2C) than of Grx1 (Fig. 2D). The IC_50_ values for the inhibition of Trx1 and Grx1 were 5.40, and 57.7 µM, respectively, which is close to the IC_50_ values for the inhibition of the whole Trx or Grx systems. This indicates that Trx1 and Grx1 are direct reactants with preheated APR246. Interestingly, the activity of Trx1 was fully restored after long-term kinetic measurements (data not shown), indicating that the initial inhibitory effect of preheated APR-246 is transient.

To examine the ability of MQ to form adducts with Trx1 and Grx1, we performed mass spectroscopy analysis. Pre-reduced Trx1 and Grx1 (50 µM) were incubated with MQ for one hour at 37 °C and then analyzed with MALDI-TOF mass spectrometry (Supplementary method). We used MQ 0.5 mM in order to override the reductant DTT in the protein preparation. The results show a clear mass shift of both Trx1 and Grx1 upon incubation with 0.5 mM MQ, indicating the formation of adducts (Supplementary Fig. 1A). Five peaks with multiple mass shifts of 137 Da, expected the molecular mass of bound MQ, were found after incubation of Trx1 with MQ at a ratio of 1:1–1:5, indicating modification of all 5 thiols at this concentration. In contrast to Trx1, incubation of Grx1 with 0.5 mM MQ resulted in 4 adducts and 1 mM MQ was needed for modification of all 5 thiols (Supplementary Fig. 1B).

### Inhibition of Trx1 and Grx1 by preheated APR-246 is thiol-dependent and reversible

**Figure 3:**
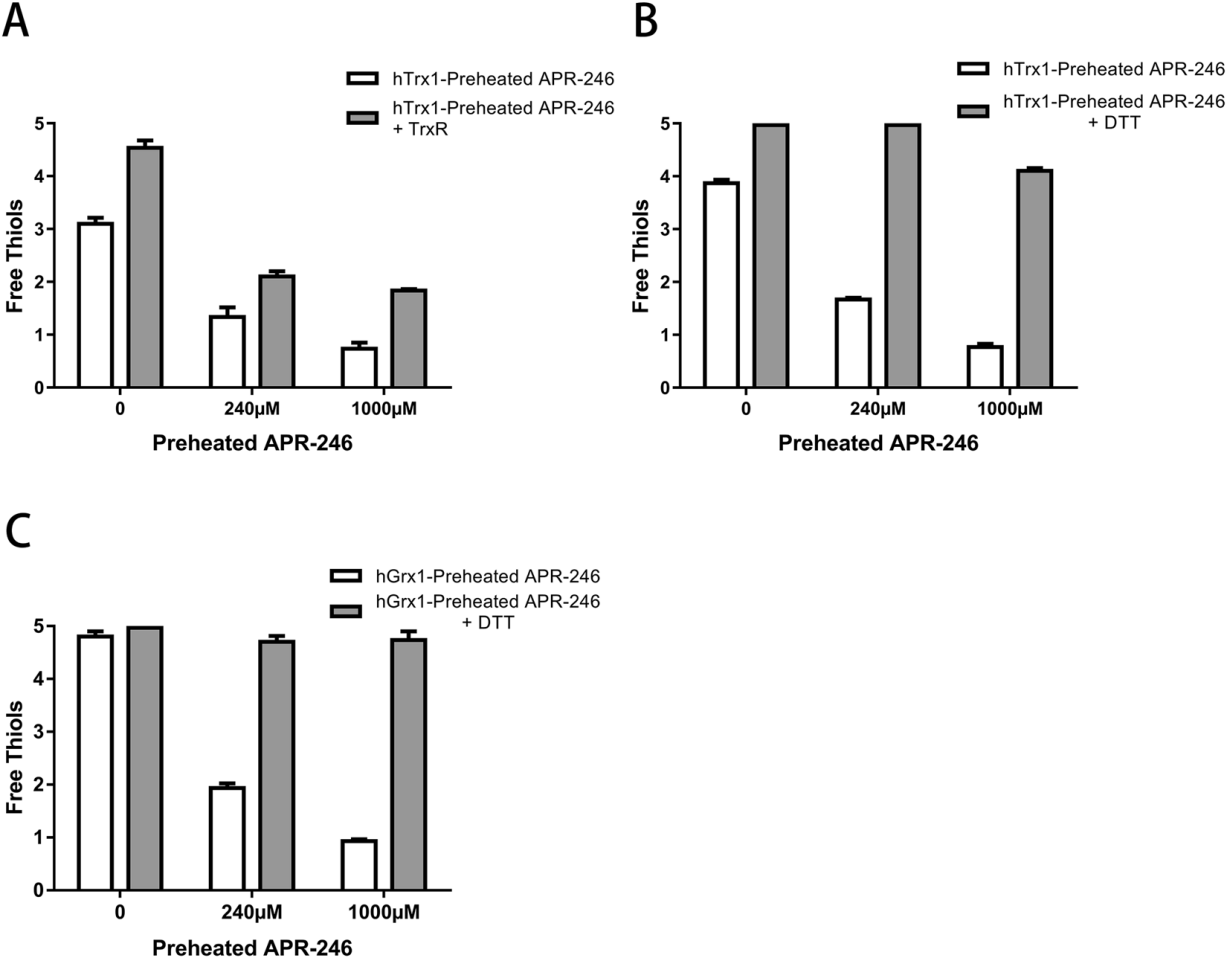
Efficient inhibition of Trx1 and Grx1 by preheated APR-246 is thiol-dependent and reversible. Indicated concentrations of preheated APR-246 efficiently inhibited 20 µM fully reduced Trx1 and the inhibition was significantly reversed by incubation with (**A**) 50 nM TrxR or (**B**) 10 mM DTT. (**C**) Inhibition of 20 µM Grx1 is significantly reversed upon incubation with 10 mM DTT. Amount of free thiols was determined with the DTNB assay^49^. Results are the means ± SEM (n = 3).

To further study the reversibility of preheated APR-246-mediated inhibition of Trx1 and Grx1, we assessed the effect on the thiols in the two proteins. To determine if they could be fully restored after preheated APR-246 inhibition, preheated APR-246-treated Trx1 was incubated with either 50 nM TrxR and 0.25 mM NADPH (Fig. 3A) or 10 mM DTT (Fig. 3B); APR-246-treated Grx1 was also incubated with 10 mM DTT (Fig. 3C). Human Trx1 and Grx1 both contain five thiols. After the incubation, there were only 3–4 thiols in the control Trx1, indicating that it got partially recovered (Fig. 3A,B). Grx1 had five thiols, demonstrating that it is in a fully reduced form (Fig. 3A). Treatment with preheated APR-246 resulted in a decrease in the amount of free thiols in both Trx1 and Grx1 in a concentration-dependent manner. The number of free thiols was substantially recovered for both Trx1 and Grx1 after reduction with DTT, indicating that the inhibition was reversed by strong reductants.

### APR-246 modifies Trx1 and Trx2 in living cells

**Figure 4:**
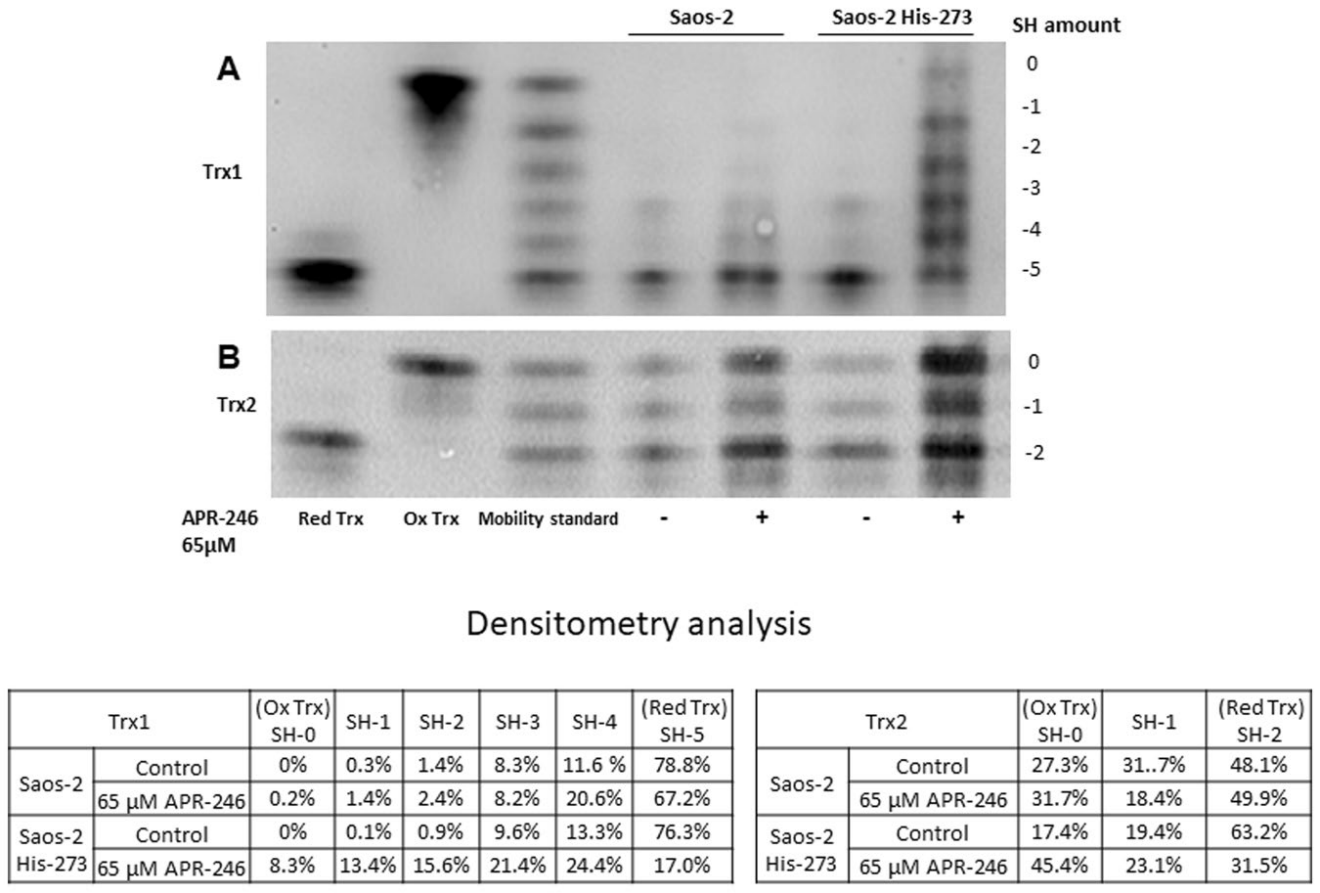
Effects of APR-246 on redox status of Trx1 and Trx2 in Saos-2 and Saos-2 His-273 cells. p53 null Saos-2 and mutant p53-expressing Saos-2-His-273 cells were treated with 65 µM APR-246 for 24 hours. The redox states of Trx1 (upper) and Trx2 (lower) were detected with a redox Western blotting method (two separated gels, original picture see Supplementary Fig. S2). The table shows the percentage of adjusted volume of each band for respective treatment groups.

To confirm the effect of APR-246 on Trx1 in living cells, we used a redox Western blot method to detect changes in the redox status of Trx1 and Trx2. Saos-2 cells, either p53 null or carrying His-273 mutant p53, were treated with 65 µM APR-246 for 24 hours prior to blotting. We observed a significant loss of free thiols in both Trx1 and Trx2 in Saos-2-His-273 cells after treatment (Fig. 4). Similar results were obtained for Saos-2 cells, although the effect was much less pronounced. Quantification of band intensities show that most Trx1 protein has free thiols in untreated Saos-2 (78.8%) and Saos-2-His-273 (76.3%) control cells (Fig. 4).

However, treatment with APR-246 resulted in a clear decrease in the amount of free thiols in mutant p53-carrying Saos-2-His-273 cells, as the amount of fully reduced thiols was decreased from 76.3% to 17.0%, and as the amount of Trx1 without any free thiols (SH-0) increased with 8%, We obtained similar results for Trx2, where almost 50% of Trx2 in Saos-2 cells was in the fully reduced form and 63% in Saos-2-His-273 cells (Fig. 4). In Saos-2 cells, APR-246 treatment led to an increase in protein without any free thiols (SH-0) from 27% to 31%, and in Saos-2-His-273 cells, the increase was even more pronounced, from 17% to 45% of Trx2 without free thiols. These changes of Trx redox status correlate with induction of cell death in Saos-2 and Saos-2-His-273 cells, as previously described^15^.

Taken together, these results show a significant inactivation of both Trx1 and Trx2 in Saos-2 and Saos-2-His-273 cells upon treatment with APR-246, through MQ binding to free thiols. This effect is more visible in Saos-2-His-273 cells than in the p53 null Saos-2 cells.

### Total glutathionylation in Saos-2 and Saos-2-His-273 cells

**Figure 5:**
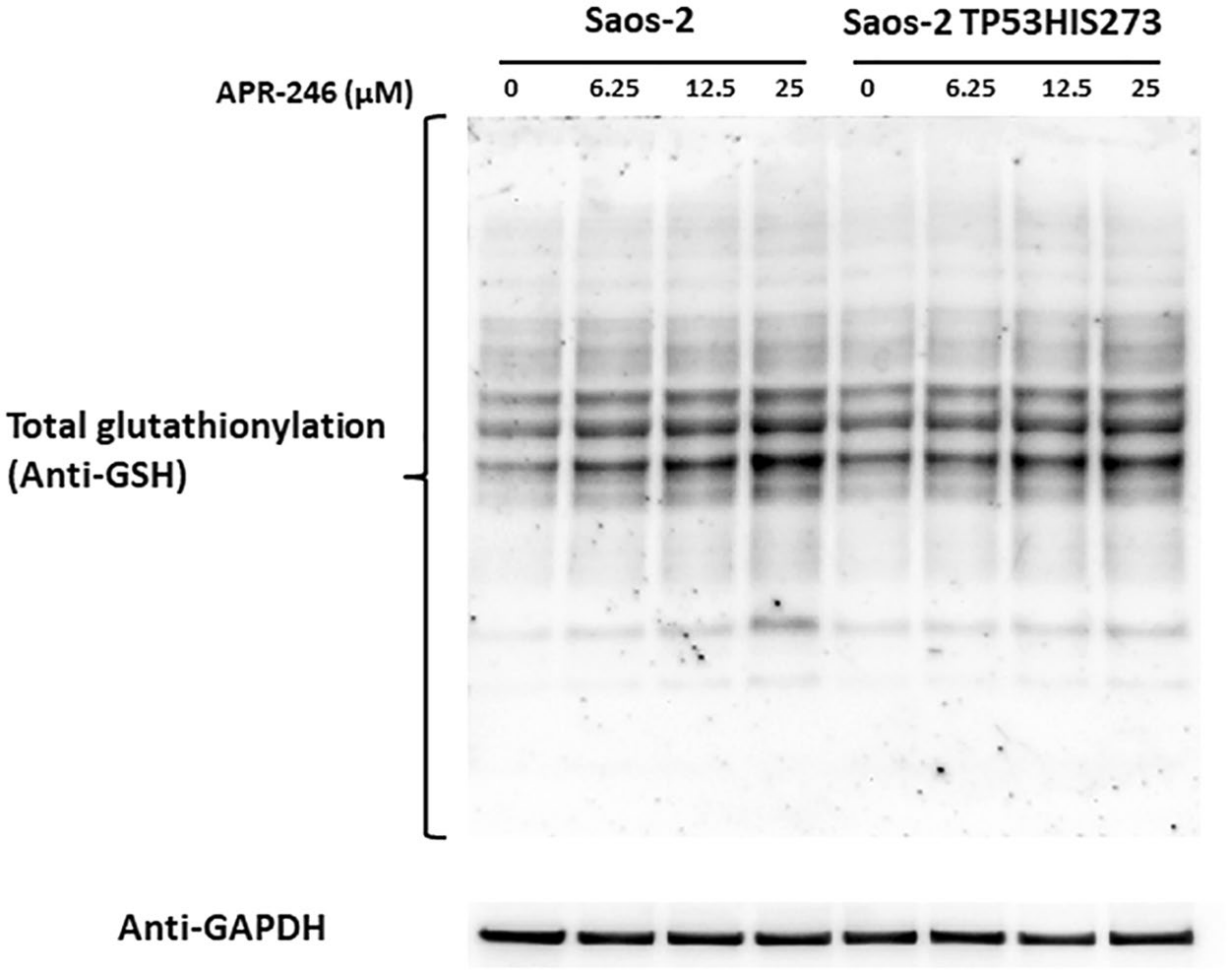
Effects of preheated APR-246 on total cellular glutathionylation in Saos-2 and Saos-2 His-273 cells. After 24 hours treatment, cells were lysed and the supernatant was separated by SDS-PAGE (12%) and probed with an anti-glutathione antibody (VIROGEN Corporation). Anti-GAPDH (Santa Cruz) antibody was used as loading control.

Protein glutathionylation is a reversible formation of mixed-disulfide between glutathione and protein thiols, which is an important event involved not only in the protection of protein cysteines from irreversible oxidation but also in protein redox regulation. Since Grx1 is involved in the deglutathionylation process and is affected by APR-246, we treated Saos-2 and Saos-2-His-273 cells with different concentrations of APR-246 for 24 hours and analyzed total glutathionylation. As shown in Fig. 5 the total glutathionylation increased with the increasing concentration of APR-246.

### APR-246 and MQ inhibit RNR activity

**Figure 6:**
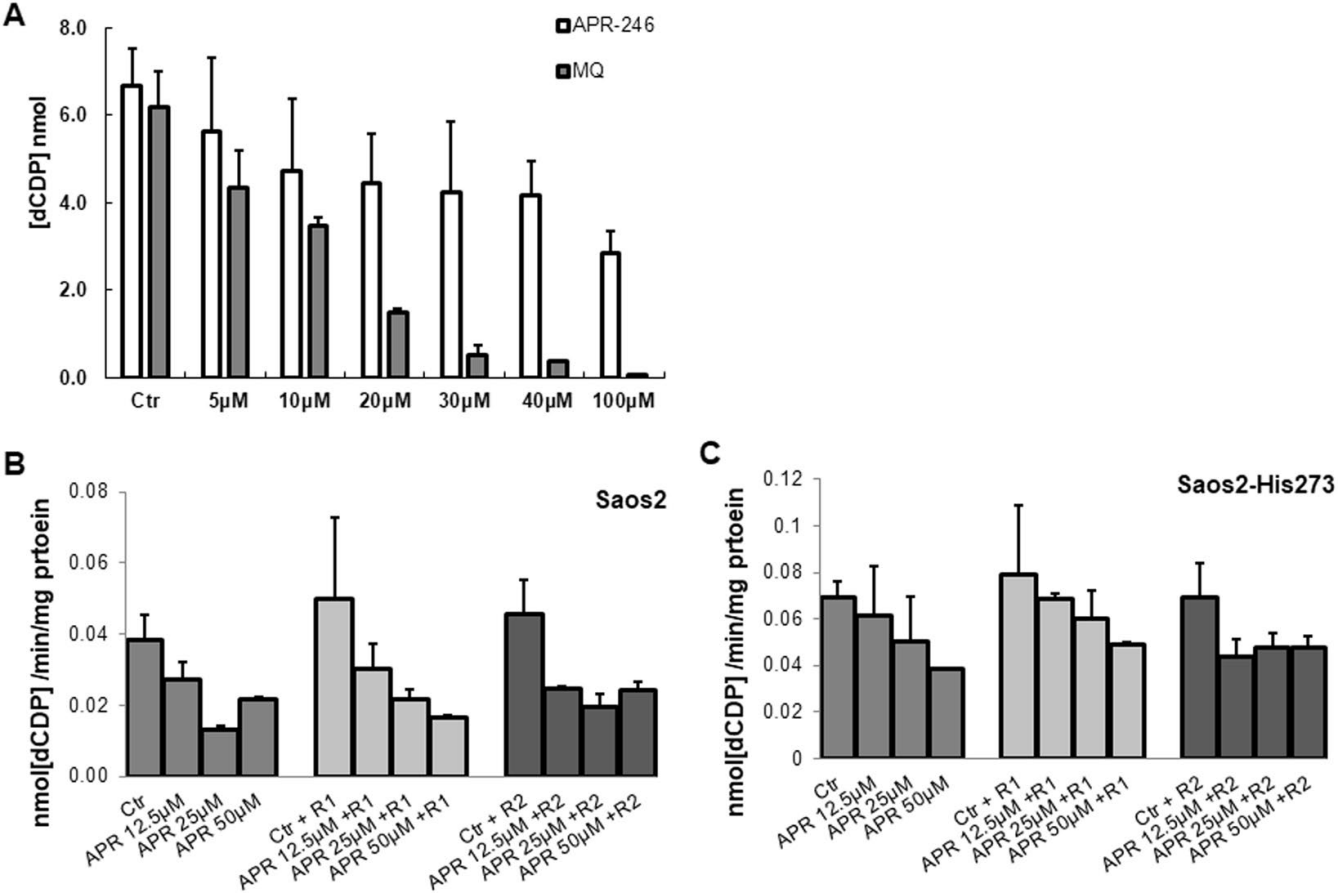
Effect of APR-246 and MQ on RNR activity. (**A**) Effects of APR-246 and MQ on RNR activity *in vitro* using recombinant R1 and R2 proteins. (**B**) RNR activity in Saos-2 cells treated with 12.5, 25 and 50 µM of APR-246 for 24 hours with addition of excess recombinant R1 and R2. (**C**) RNR activity in Saos-2-His273 cells treated with 12.5, 25 and 50 µM of APR-246 for 24 hours with addition of excess recombinant R1 and R2.

In order to test whether APR-246 and MQ have a direct effect on ribonucleotide reductase (RNR) apart from Trx and Grx, we assessed the effect of APR-246 and MQ on RNR activity both *in vitro* and in cells. Both APR-246 and MQ inhibited RNR activity *in vitro*, but as expected, MQ was significantly more potent. MQ at 40 µM almost completely inhibited the activity (Fig. 6A). We also treated Saos-2 and Saos-2-His-273 cells with 12.5, 25, and 50 µM of APR246 for 24 hours at 37 °C and assessed RNR activity in the cell lysates. This revealed a dose-dependent inhibition of RNR by APR-246 in both Saos-2 p53 null and Saos-2-His-273 cells.

## Discussion

The Trx and Grx systems are both commonly upregulated in tumor cells to compensate for the enhanced ROS production as a result of increased metabolism. The Trx system provides electrons to peroxiredoxins and methionine sulfoxide reductases (MSR) to protect cells against oxidative stress^34^. Either Trx1 or Grx1 are required for each turnover of RNR, the essential enzyme for deoxyribonucleotides synthesis during DNA replication and repair. Both redoxins are also known to have anti-apoptotic properties through interactions with several transcription factors and proteins, such as inhibition of the MAPK cascade by binding to ASK-1 in their reduced state^24,35^.

Thessoulin and colleagues have shown that APR-246 (PRIMA-1^Met^) induces cell death in myeloma cells by GSH depletion^17^. We have shown that MQ can bind GSH and decrease GSH levels in ovarian cancer cells^16^. Since MQ will consume free GSH, all pressure in the cell to prevent oxidative stress will be put on the Trx system. However, TrxR is inactivated by MQ and converted to an NADPH oxidase that produces ROS^15^. Although GSH may act as a backup for TrxR to keep Trx in its reduced form^36^, it will be depleted by APR-246, leading to the dramatically increased ROS production due to the simultaneous inactivation of both major redox systems. If the cell contains mutant p53, APR-246 will reactivate mutant p53 and induce pro-apoptotic p53 target genes, which may lead to a further increase in ROS^37^.

We also observed a significant loss of free thiols in both Trx1 and Trx2 in APR-246-treated Saos-2-His-273 cells. The effect in p53 null Saos-2 cells was much less pronounced than in the mutant p53-expressing cells. It is likely that reactivation of overexpressed mutant p53 by APR-246, along with APR-246-mediated inhibition of TrxR1, leads to increased production of ROS, which makes Trx1 oxidized. Via peroxiredoxin, H_2_O_2_ can inactive Trx1 forming a two-disulfide form^38^. Furthermore, mutant p53 has been shown to inhibit the activity of the antioxidant transcription factor Nrf2, which may lead to reduced expression of SLC7A11, a component of the x_c_ cystine-glutamate antiporter, and thus decreased levels of GSH^39^. This provides an additional plausible explanation for the stronger effect on thiols in Trx1 and Trx2 in the mutant p53-expressing Saos-2 cells. The oxidation of Trx can lead to non-repaired DNA damage by loss of RNR activity, activate the MAPK pathway via ASK1, and further induce ROS production^40^. In combination with reactivation of mutant p53 by APR-246, the oxidation of Trx might facilitate p53-dependent apoptosis.

The depletion of GSH in the APR-246 treated cells was also confirmed by the result that increased total glutathionylation was observed in both cell lines in a dose-dependent manner. The increased glutathionylation is a sign of oxidative stress in the cells and is probably due to inhibition of Grx1 which can efficiently catalyze protein deglutathionylation (Fig. 5).

Thioredoxin and glutaredoxin systems provide the reducing power to RNR for balanced deoxyribonucleotide pools for DNA replication and repair. We carried out an *in vitro* RNR assay to test whether APR-246 via MQ has a direct effect on RNR activity. We found that both APR-246 and MQ inhibited RNR activity, with MQ being more potent (Fig. 6A). The ability of APR-246 itself to inhibit RNR in this experiment may be due to the generation of MQ in the APR-246 preparation. We also found that APR-246 inhibits RNR activity in Saos-2 cells. The inhibition was more potent in p53 null Saos-2 p53 cells (Fig. 6B,C).

In order to understand the mechanism for APR-246-induced loss of the thiols in Trx and Grx, we performed mass spectrometry analyses, which revealed direct binding of MQ to Trx1 and Grx1. These results provide solid confirmation that the decrease in thiol content observed in the proteins is indeed due to the formation of MQ adducts.

Inhibition of the thioredoxin system has been proposed as a novel anticancer approach^34,40,41^. Many compounds and clinical drugs have been shown to inhibit TrxR and lead to subsequent Trx oxidation^40,42,43^. Some Michael acceptor drugs can inactivate and convert the reductant TrxR into a pro-oxidant to cause the oxidation of Trx, the key determining factor for the cell fate^38,40,44^. APR-246 may also act in the same manner^15^.

Our analysis of Trx and Grx using redox Western blotting provided information about the fraction of each protein in the different redox states in the cells after APR-246 treatment. Human Trx1 contains two cysteines, Cys32 and Cys35, in the active site motif Trp-Cys-Gly-Pro-Cys, with three additional structural cysteines (Cys62, Cys69, and Cys73). After electron transfer to its substrate via the dithiol-disulfide exchange reaction, a disulfide bond is formed between Cys32 and Cys35, which is reduced by TrxR and NADPH. The three structural cysteines can be modified by glutathionylation or nitrosylation^38^. Cys73 may also be involved in Trx1 dimer formation through a Cys73-Cys73 disulfide bond. However, since our data show an inhibition of Trx activity by APR-246, there is a great probability that the shift in redox states observed in the redox Western blots also are results of MQ modifications. Loss of free thiols in Trx1 leads to the release of ASK1, which activates the c-Jun N-terminal kinase (JNK) and p38 MAP kinase pathways and in turn TNF-α-induced apoptosis^24^. The APR-246 structural analog PRIMA-1 has been shown to induce apoptosis through the JNK pathway, which synergizes with the apoptotic pathway induced by Trx1 oxidation via ASK1 release^45^. Thus, it is possible that the induction of the MAP kinase pathway by MQ could be a result of Trx1 inactivation, as shown in this study. Trx2 also induces apoptosis via ASK1 when being oxidized, as well as giving rise to increased ROS levels in the mitochondria, leading to release of cytochrome c and activation of caspase 3 and 9^46^. Interestingly, the APR-246-induced cell death was in part caspase 2-independent^45^. Our findings indicate that inhibition of Trx2 by APR-246 can be the explanation for this effect^47^.

A recent paper demonstrated that PARP-1 inhibitors sensitized head and neck squamous cell carcinoma cells to APR-246 by inactivation of TrxR and production of ROS^48^. The results showed that APR-246 may kill cancer cells independent of p53^48^.

In conclusion, we have shown that APR-246 reversibly inhibits Trx, Grx, and RNR, most likely through covalent binding of MQ to thiols. This, together with the previously reported inactivation of TrxR via modification of the selenocysteine residue in the active site and the fast reaction with GSH, will disrupt the redox balance in the cell and affect processes such as DNA replication and repair and neutralization of ROS. Thus, APR-246 targets both mutant p53 and cellular redox regulation, two Achilles’ heels of tumor cells. Our data shed novel light on the mechanisms behind APR-246-induced apoptosis in cancer cells. Given that APR-246 is currently in clinical development, these results may have implications for its use as an anticancer agent.

## Electronic supplementary material

Supplementary Information
