# Supplementary material for: Inhibition of the glutaredoxin and thioredoxin systems and ribonucleotide reductase by mutant p53-targeting compound APR-246
Source: Sci Rep. 2018 Aug 23;8:12671. doi: 10.1038/s41598-018-31048-7 (PMC6107631; doi:10.1038/s41598-018-31048-7)
Supplement: Supplementary file 1 — Supplementary Information [file 41598_2018_31048_MOESM1_ESM.pdf]

# Inhibition of the glutaredoxin and thioredoxin systems and ribonucleotide reductase by mutant p53-targeting compound APR-246

Lena Haffo[a,b,1], Jun Lu[a,1], Vladimir J.N. Bykov[b], Sebastin S Martin[a], Xiaoyuan Ren[a], Lucia Coppo[a], Klas G. Wiman[b,*], Arne Holmgren[a,*]

[a]Division of Biochemistry, Department of Medical Biochemistry and Biophysics, Karolinska Institutet, SE-171 77, Stockholm, Sweden

[b]Department of Oncology-Pathology, Cancer Center Karolinska (CCK), Karolinska Institutet, SE-171 76 Stockholm, Sweden

Running title: Inhibition of Trx, Grx and ribonucleotide reductase by APR-246

Keywords: thioredoxin, glutaredoxin, ribonucleotide reductase, p53, APR-246, reactive oxygen species

Chemical compounds studied in this article: APR-246 (PubChem CID: 52918385); Methylene quinuclidinone (PubChem CID: 16219655)

*Corresponding authors:

Prof. Arne Holmgren, Division of Biochemistry, Department of Medical Biochemistry and Biophysics, Karolinska Institute, SE-171 77 Stockholm, Sweden, Tel: +46 8 52487686; Fax: +46 8 7284716; Email: arne.holmgren@ki.se

Prof. Klas G. Wiman, Department of Oncology-Pathology, Cancer Center Karolinska (CCK), Karolinska Institutet, SE-171 76 Stockholm, Sweden, Tel: +46 8 5177 9342; Email: Klas.Wiman@ki.se

[1] These authors equally contributed to this work.

Supplementary material

## Method

### *1.1. Mass spectrometry analysis*

Pre-reduced recombinant hTrx1 and hGrx1 (50 µM) were incubated with 0.1 and 0.5mM MQ, respectively, for one hour at 37°C. Modified proteins were desalted with C4 ZipTip and then crystallized on a matrix-assisted laser desorption/ionization (MALDI) target plate with MALDI matrix prepared through dissolving 10 mg Sinapinic acid in 75% acetonitrile and 0.1% trifluoroacetic acid (TFA). MQ-modified Trx1 and Grx1 were identified using MALDI-TOF mass spectrometry (Voyager DE-Pro, AB SCIEX). Each spectrum was the result of 50 laser shots and myoglobin was used for external calibration.

## Supplementary Result

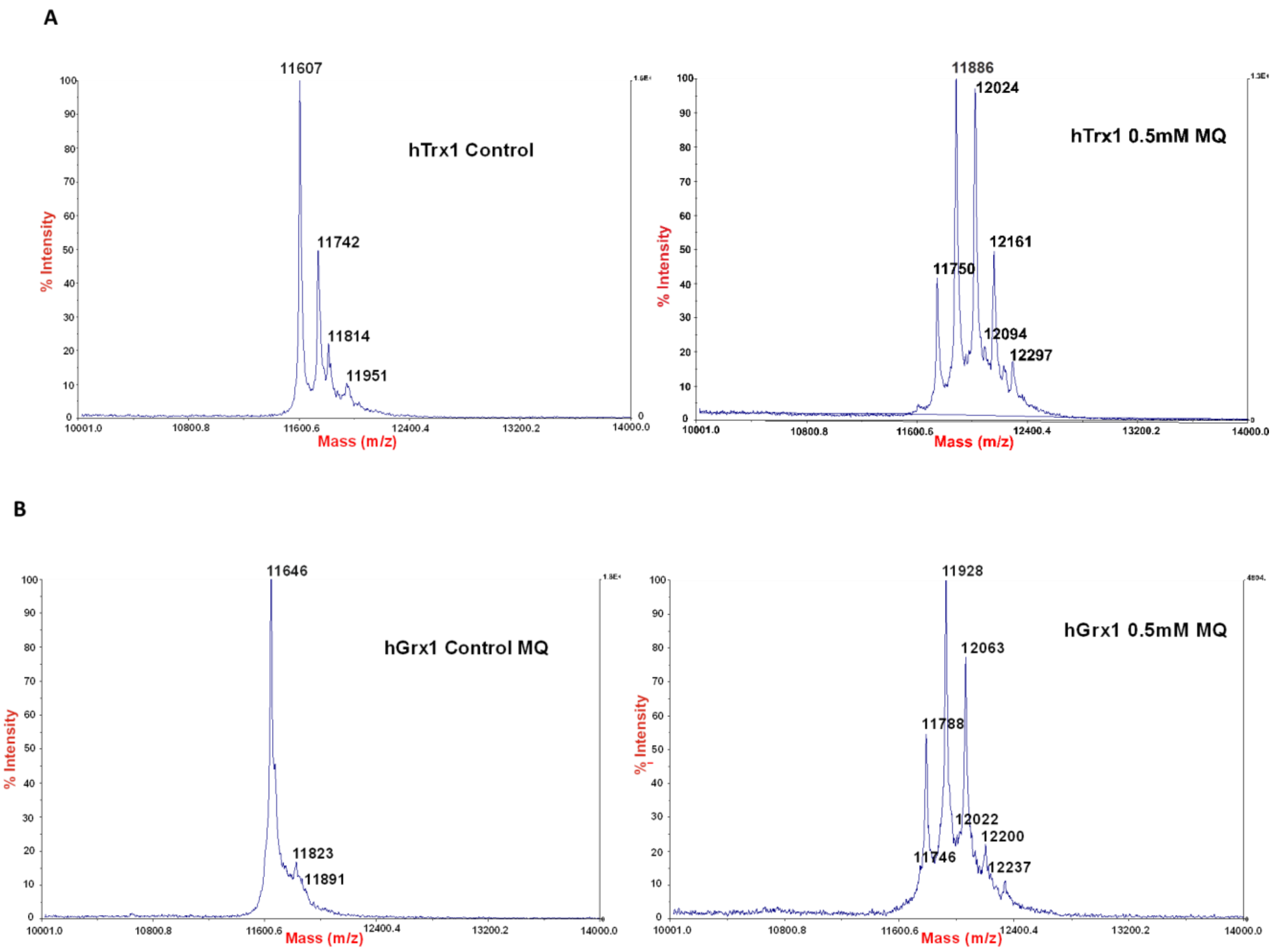


**Figure S1**. **Interactions of human Trx1/Grx1 and MQ analyzed by MALDI-TOF mass spectrometry.** (A) Reduced hTrx1(left panel) was incubated with 0.5 mM MQ (right panel), mass shift was shown with multiple MQ adducts, indicating all 5 cysteines was modified by MQ. (B) Reduced hGrx1 (left panel) was incubated with 0.5 mM MQ (right panel), 4 adducts were seen in mass spectrum.

**A**

Redox western blot of Trx1

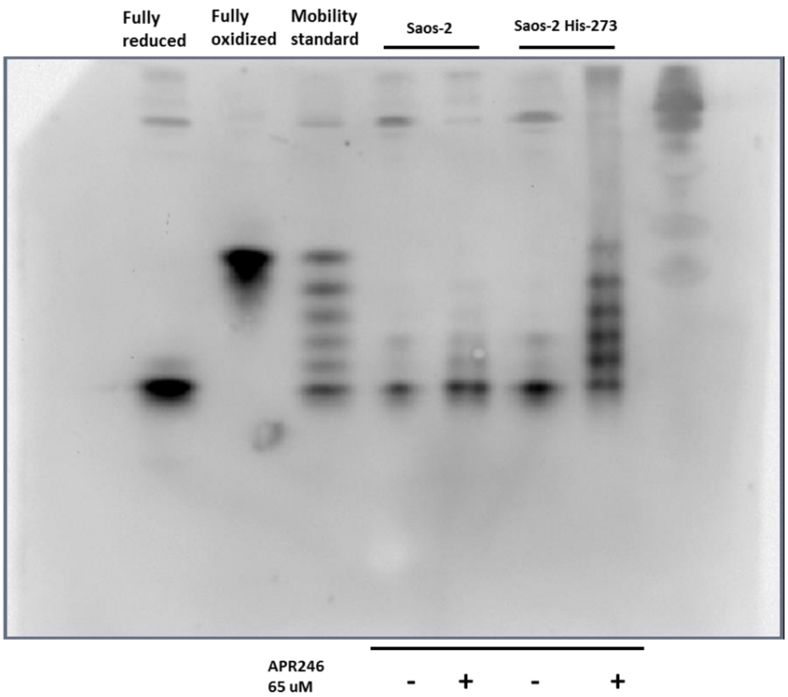


**B**

Redox western blot of Trx2

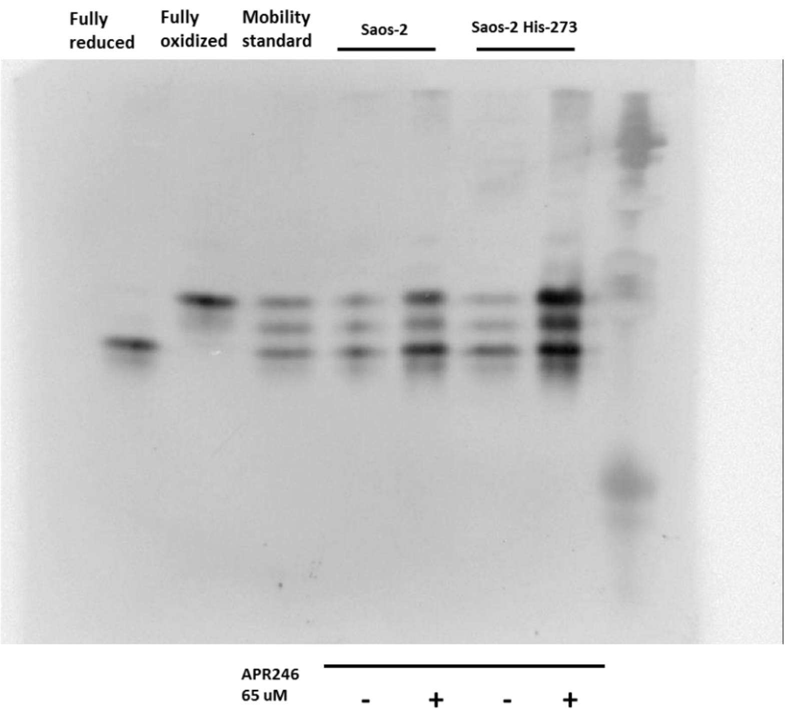

**Figure S2. Effects of APR-246 on redox status of Trx1 and Trx2 in Saos-2 and Saos-2 His-273 cells.** p53 null Saos-2 and mutant p53-expressing Saos-2-His-273 cells were treated with 65 μM APR-246 for 24 hours. The redox states of Trx1 (A) and Trx2 (B) were detected with a redox Western blotting method.
